# Highly Efficient Labeling of Human Lung Cancer Cells Using Cationic Poly-l-lysine-Assisted Magnetic Iron Oxide Nanoparticles

**DOI:** 10.1007/s40820-015-0053-5

**Published:** 2015-07-16

**Authors:** Xueqin Wang, Huiru Zhang, Hongjuan Jing, Liuqing Cui

**Affiliations:** College of Bioengineering, Henan University of Technology, Zhengzhou, 450001 Henan People’s Republic of China

**Keywords:** Magnetic labeling, Iron oxide nanoparticles, Poly-l-lysine, Human A549 lung cancer cells, Cancer treatment

## Abstract

Cell labeling with magnetic iron oxide nanoparticles (IONPs) is increasingly a routine approach in the cell-based cancer treatment. However, cell labeling with magnetic IONPs and their leading effects on the biological properties of human lung carcinoma cells remain scarcely reported. Therefore, in the present study the magnetic γ-Fe_2_O_3_ nanoparticles (MNPs) were firstly synthesized and surface-modified with cationic poly-l-lysine (PLL) to construct the PLL-MNPs, which were then used to magnetically label human A549 lung cancer cells. Cell viability and proliferation were evaluated with propidium iodide/fluorescein diacetate double staining and standard 3-(4,5-dimethylthiazol-2-diphenyl-tetrazolium) bromide assay, and the cytoskeleton was immunocytochemically stained. The cell cycle of the PLL-MNP-labeled A549 lung cancer cells was analyzed using flow cytometry. Apoptotic cells were fluorescently analyzed with nuclear-specific staining after the PLL-MNP labeling. The results showed that the constructed PLL-MNPs efficiently magnetically labeled A549 lung cancer cells and that, at low concentrations, labeling did not affect cellular viability, proliferation capability, cell cycle, and apoptosis. Furthermore, the cytoskeleton in the treated cells was detected intact in comparison with the untreated counterparts. However, the results also showed that at high concentration (400 µg mL^−1^), the PLL-MNPs would slightly impair cell viability, proliferation, cell cycle, and apoptosis and disrupt the cytoskeleton in the treated A549 lung cancer cells. Therefore, the present results indicated that the PLL-MNPs at adequate concentrations can be efficiently used for labeling A549 lung cancer cells and could be considered as a feasible approach for magnetic targeted anti-cancer drug/gene delivery, targeted diagnosis, and therapy in lung cancer treatment.

## Introduction

Lung cancer is the most common cause of cancer-related deaths worldwide [1, 2]. According to the US National Cancer Institute, approximately 226, 160 new lung cancers were diagnosed and 160, 340 lung cancer-related deaths were recorded in the US at the end of 2012. Despite advances in cancer research, the discovery of lung cancer biomarkers has not significantly improved conventional treatments in recent years [3]. Therefore, the development of novel approaches for the detection of early lung cancer-specific markers for personalized treatment is urgently needed to increase patient survival in lung cancer treatment. Nanotechnology has provided new perspectives for molecular detection, cellular imaging, and screening in cancer treatment. Despite the fact that nanotechnology can provide sensitive and specific information from lung cancer patients, the selective delivery of anti-cancer drugs to tumor sites in lung cancer treatment is also highly desirable [4, 5].

Magnetic nanoparticles (IONPs) (mostly γ-Fe_2_O_3_ and Fe_3_O_4_) have received an increasing interest in biomedical applications because of their low toxicity, outstanding biocompatibility, and superparamagnetic properties applicable in cell labeling and separation [6, 7], cell imaging [8, 9], cell-based therapy [10, 11], magnetofection [12], and tissue engineering and repair [13, 14], among other uses. Further, IONPs have a large surface area engineered with a number of functional groups capable of cross-linking to tumor-targeting ligands, such as antibodies, peptides, or small molecules, for diagnostic imaging or delivery of therapeutic agents [15–17]. IONPs for cell labeling have been widely used to isolate cells of interest from a heterogeneous population within a magnetic field via phenotypic markers tagged with magnetic particles [18, 19]. The labeling of cells with IONPs allows to noninvasively monitor the migration, biodistribution, and behavior of targeted cells. Therefore, the efficient magnetic labeling of targeted cells offers promising new approaches in cell-based therapy with great potential for cancer treatment [20].

The magnetic labeling of cells can be performed following either of two approaches: (i) the direct attachment of magnetic particles to the cell surface [21] or (ii) the receptor-mediated or transfection agent-mediated endocytosis pathway which internalizes biocompatible magnetic particles [16]. A variety of competent ligands and target agents conjugated onto nanoparticle surfaces, including monoclonal antibodies, transferrin, and folic acid, have been used to target cell surfaces [22–24]. The endocytosis pathway, which involves the transfection of IONPs into cells, requires a high rate of nanoparticle internalization by the cells. However, both the cell membrane and IONPs are usually negatively charged, leading to the inefficient labeling of targeted cells because of repulsive electric interactions. Therefore, the complexing of positively charged transfection agents, such as polyethylenimine [25, 26], lipofectamine [27, 28], poly-l-lysine (PLL) [29, 30], and protamine sulfate [31, 32], is often introduced to enhance cell labeling efficiency through favorable electrostatic interactions between the target cells and transfection agent-modified IONPs. PLL is commonly used to enhance cell adhesion on the surface of culture dishes and is a potent modifier for IONPs [33]. A PLL coating on IONPs could lead to improved electrostatic binding on target cells and thus enhance cellular uptake.

In the present study, the PLL-modified MNPs were constructed to label human A549 lung cancer cells. Compared with previous studies [34, 35], the present study attempted to use cationic transfection agent PLL coupled with MNPs to enhance the cellular uptake of nanoparticles in human A549 lung cancer cells. Furthermore, the effects of magnetic labeling on the biological properties of A549 lung cancer cells, including cell viability, proliferation capacity, cytoskeletal disruption, cell cycle, and apoptosis, were investigated separately. The morphology and structure of the PLL-MNPs were characterized by transmission electron microscopy (TEM), X-ray diffraction (XRD), Fourier transform infrared spectroscopy (FT-IR), and vibrating sample magnetometry. The iron uptake of magnetically labeled A549 lung cancer cells was confirmed with histological Prussian blue staining. The viability of magnetically labeled A549 lung cancer cells was assessed by fluorescein diacetate (FDA) and propidium iodide (PI) double staining. The cytoskeletal disruption and apoptosis rate of magnetically labeled A549 lung cancer cells was assayed by rhodamine-conjugated phalloidin and fluorescent bisbenzimide dye (Hoechst H33258). The cell cycle of the labeled A549 lung cancer cells was analyzed by flow cytometry. Taken together, this study provides insights into the effects of magnetic labeling on the biological properties of A549 lung cancer cells and facilitates the MRI-assisted tracking and in vivo monitoring of their survival, migration, and transformation, which would be of great interest for targeted lung cancer cell treatment.

## Experimental

### Reagents and Materials

The superparamagnetic γ-Fe_2_O_3_ NPs utilized in this study were prepared from magnetite (Fe_3_O_4_) according to the methods proposed elsewhere [36, 37]. The human lung alveolar carcinoma epithelial cells (A549) were obtained from the Shanghai Cell Bank of the Chinese Academy of Sciences (Shanghai, China). The cell culture medium and fetal bovine serum (FBS) were purchased from Gibco Invitrogen Corporation (CA, USA). PLL (*M*_w_ = 338,100), 3-(4,5-dimethylthiazol-2-diphenyl-tetrazolium) bromide (MTT), potassium ferrocyanide (Perls reagent), FDA, PI, RNase, Hoechst H33258, dimethyl sulfoxide (DMSO), Triton X-100 solution, glutaraldehyde, and paraformaldehyde were purchased from Sigma-Aldrich (St. Louis, MO, USA). Neutral red was obtained from Beyotime Biotech (Jiangsu, China). Fluorescent dye 4,6-diamidino-2-phenylindole (DAPI) was purchased from Molecular Probes Inc. (Eugene, OR, USA). Rhodamine phalloidin was obtained from Cytoskeleton Inc. (Denver, CO, USA). Other reagents and chemicals were purchased from local commercial suppliers and were of analytical reagent grade, unless otherwise stated. Deionized water (Milli-Q, Millipore, Bedford, MA, USA) was used to prepare aqueous solutions.

### Principle of A549 Lung Cancer Cell Labeling with PLL-Modified MNPs

**Scheme 1:**
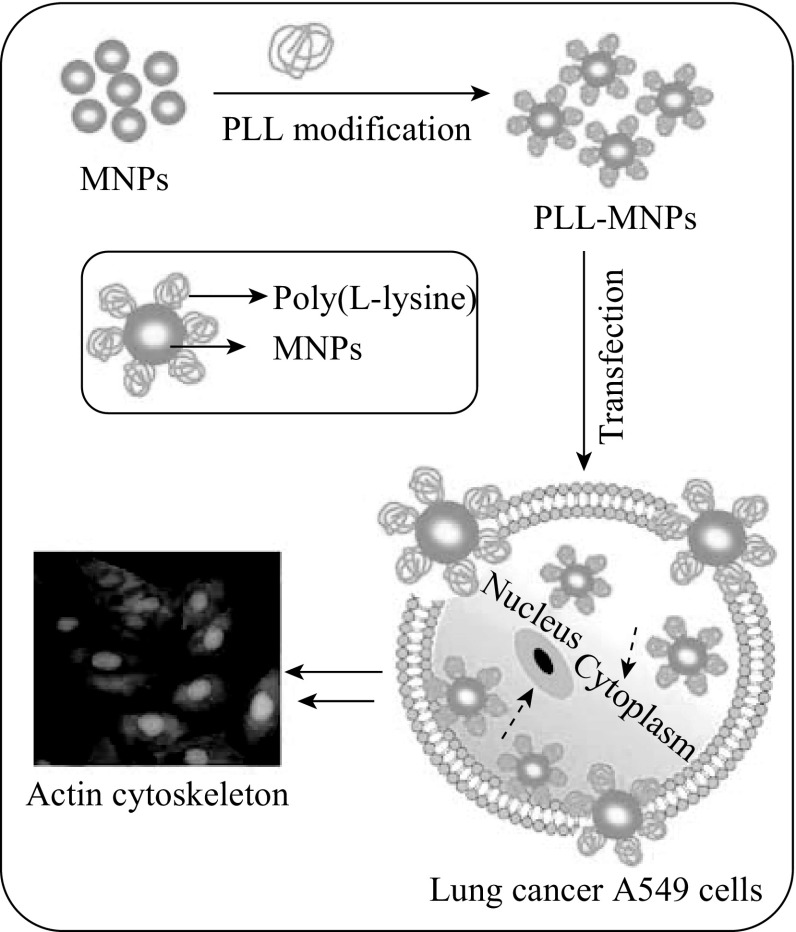
Schematic representation of the PLL-MNP preparation and delivery to human A549 lung cancer cells

Following modification of magnetic γ-Fe_2_O_3_ nanoparticles (MNPs) with PLL to create the PLL-MNPs, the latter was putatively delivered to human A549 lung cancer cells (Scheme 1). Subsequently, the effects of the PLL-MNPs on the biological properties of A549 lung cancer cells, including proliferation capacity, cell viability, actin cytoskeleton disruption, and apoptosis rate, were further investigated after magnetic labeling. Therefore, this work would clearly elaborate the effects of magnetic labeling on biological properties of lung cancer cells using the constructed PLL-MNPs, facilitating IONP-assisted targeted drug and/or gene delivery in cancer treatment.

### Synthesis and Characterization of the PLL-MNPs

MNPs used in the study were prepared and characterized as previously described elsewhere [36, 37], and the PLL modification was then performed to enhance cellular uptake to NPs [38]. Briefly, 3.6 mL of MNPs (2 mg mL^−1^) were mixed with 3.6 mL of serum-free RPMI-1640 medium, followed by the addition of 0.8 mL of PLL (0.45 mg mL^−1^). The solution containing MNPs and PLL was then incubated with low-speed stirring for 2 h at 200 rpm, and the prepared PLL-modified MNPs were washed to remove residual PLL with serum-free RPMI-1640 medium under the magnetic field for three times. Finally, the PLL-MNPs were vacuum desiccated and stored at 4 °C until further use.

The morphology of MNPs and PLL-MNPs was characterized by TEM using a Hitachi H-600 microscope (Japan). The crystal structure of MNPs was characterized by an X-ray diffractometer (Philips D/Max-2500, Holland) using a monochromatized X-ray beam with nickel-filtered Cu-*Kα* radiation. FT-IR (Nicolet NEXUS 670, USA) was performed to record the spectra of MNPs and PLL-MNPs. Magnetic measurements of the PLL-MNPs were performed on a vibrating sample magnetometer (Lakeshore-7304, USA) by changing *H* between +1375 and −1375 Oe.

### Cell Culture

The human A549 lung cancer cells were routinely cultured in RPMI-1640 medium supplemented with 10 % (v/v) heat-inactivated FBS, 1 % l-glutamine, 1 % penicillin (100 U mL^−1^), and 1 % streptomycin (100 µg mL^−1^) in a humidified incubator at 37 °C in the presence of 5 % CO_2_. The cells were regularly monitored using an inverted light microscope, and the culture medium was changed every 2 days. The cells were normally passaged in 1:3 ratios every 3 days to maintain an exponential growth phase.

### Labeling of A549 Lung Cancer Cells with the PLL-MNPs

The A549 cells at the exponential growth phase were treated with 0.25 % trypsin in Ca^+^- and Mg^+^-free phosphate-buffered saline (PBS, pH 7.4) for 5 min at 37 °C to prepare a cell suspension. The cells were counted using a regular hemocytometer and then seeded at a density of 2 × 10^4^ cells/well in a 24-well plate. Different concentrations of the PLL-MNPs were then added to the 24-well plates separately. The culture medium was discarded after incubation for 48 h, and the PLL-MNP-labeled A549 cells were then incubated with a mixture (50:50, v/v) of 2 % potassium ferrocyanide (Perls reagent) and 2 % hydrochloric acid for 30 min following fixation with 4 % paraformaldehyde. Counterstaining was performed by incubating the cells with neutral red for 2 min. Unlabeled A549 cells were used as the control.

### A549 Cell Proliferation Capacity Assay after PLL-MNP Labeling

The standard MTT method was used to evaluate the proliferation capacity of A549 lung cancer cells after PLL-MNP labeling. The A549 cells were harvested to prepare a cell suspension at the exponential growth phase and then seeded at a density of 1 × 10^4^ cells/well in a 96-well microwell plate. The PLL-MNPs were added to the microwell plates at final concentrations ranging from 25 to 400 µg mL^−1^. The culture medium was removed after incubation for 48 h and 200 µL of the prepared MTT solution (final concentration: 0.5 mg mL^−1^) was then added and incubated for 4 h, followed by the addition of 150 µL DMSO. Finally, the absorbance of the prepared solutions was measured at 570 nm on a microplate spectrophotometer (Bio Tek Instrument Inc., USA). Unlabeled A549 cells were used as the control.

### A549 Lung Cancer Cell Viability Assay after PLL-MNP Labeling

The cell viability of PLL-MNP-labeled A549 cells was assessed by the FDA and PI double-staining protocol [39, 40]. The A549 cells were seeded and incubated for 12 h at a density of 2 × 10^4^ cells/well in a 24-well plate. The PLL-MNPs were added to the plate at final concentrations ranging from 25 to 400 µg mL^−1^. Subsequently, the FDA (final concentration: 1 μg mL^−1^) and PI solutions (final concentration: 20 μg mL^−1^) were successively introduced into the culture plates. The cellular viability was then analyzed by counting the live and dead cells after incubation for 10 min at room temperature. The living cells were stained green by FDA, whereas the dead cells were stained red by the fluorescent PI dye. The samples were analyzed with an inverted fluorescence microscope equipped with a high-resolution CCD camera. Unlabeled A549 cells were used as the control.

### Immunofluorescent Staining of Actin Cytoskeleton

The morphology and F-actin cytoskeletal structure of A549 cells exposed to varying concentrations of the PLL-MNPs were investigated. Actin cytoskeleton staining was performed using rhodamine phalloidin following the kit’s protocol with minor modifications. Briefly, the A549 cells were seeded in the PLL-coated 24-well plates, cultured for 12 h, and then treated with the PLL-MNPs in an RPMI-1640 medium. The PLL-MNP-labeled A549 cells were then fixed with 2.5 % glutaraldehyde for 10 min at room temperature, and 0.5 % Triton X-100 solution in PBS was introduced to permeabilize the cells by culturing for 5 min at room temperature. The permeabilized cells were washed with PBS (pH 7.4) for three times and then refilled with 200 µL of 100 nM rhodamine phalloidin. Sequentially, the cells were incubated in the dark for 30 min at room temperature. The cells were counterstained with DAPI dye to reveal nuclei. Finally, the samples were observed under an inverted fluorescence microscope equipped with a high-resolution CCD camera.

### Cell Cycle Analysis of PLL-MNP-Labeled A549 Lung Cancer Cells

The cell cycle of PLL-MNP-labeled A549 cells was analyzed by flow cytometry. The PLL-MNP- treated A549 cells were firstly harvested and dissociated into a single-cell suspension, and 1 × 10^6^ cells were resuspended in 500 µL of PBS solution after washing three times. The A549 cells were then fixed by adding 2 mL of 70 % ice-cold ethanol, followed by overnight incubation at 4 °C. The prepared cell suspension was then centrifuged to discard the fixative, resuspended in 2 mL of PBS solution, and filtered using a 200-mesh cell screen. The pelleted cells were resuspended in 1 mL of 50 µg mL^−1^ PI solution containing 20 µg mL^−1^ RNase for cell staining. The A549 cells were incubated in the dark at 4 °C for 1 h, analyzed using a FACSCalibur flow cytometer (BD Biosciences, San Jose, CA), and analyzed using CELLQUEST software (BD Biosciences). The untreated A549 cells were used as controls.

### Fluorescent Staining of Apoptotic Cells

To visualize apoptotic cells, the PLL-MNP-labeled A549 cells were fixed with 4 % paraformaldehyde for 15 min and then stained with Hoechst H33258 solution (concentration: 2 µg mL^−1^) for 10 min at room temperature. The stained cells were rinsed with PBS three times and then observed via an inverted fluorescence microscope equipped with a high-resolution CCD camera. Untreated A549 cells were used as the control.

### Instruments

A transmission electron microscope (TEM, Hitachi H-600, Japan) was employed to characterize the morphology and structure of the prepared MNPs and PLL-MNPs. The crystal structure of MNPs was analyzed with an X-ray diffractometer (XRD, Philips D/Max-2500, Holland) using a monochromatic X-ray beam with nickel-filtered Cu-*Kα* radiation. Magnetic measurements of PLL-MNPs were carried out on a vibrating sample magnetometer (LAKESHORE-7304, USA) by changing *H* between +1375 and −1375 Oe. Fluorescence images were acquired using an inverted fluorescence microscope (Eclipse TE 2000-U, Nikon, Kyoto, Japan) equipped with a high-resolution CCD camera (CV-S3200, JAI Co., Japan). The cell cycle of the PLL-MNP-labeled A549 cells were analyzed using FACS Calibur flow cytometer (BD Biosciences, San Jose, CA).

### Image Acquisition and Analysis

Bright-field and fluorescence images were acquired using an inverted fluorescence microscope (Eclipse TE 2000-U, Nikon, Kyoto, Japan) equipped with a CCD camera (CV-S3200, JAI Co., Japan). Software Image-Pro Plus^®^ 6.0 (Media Cyternetics) and SPSS 12.0 (SPSS Inc.) were used to perform image analysis and statistical data analysis, respectively. The quantitative data are presented as mean ± standard deviation for each experiment. All experiments were performed with at least three replicates, and the results presented were obtained from representative experiments.

## Results and Discussion

### Synthesis and Characterization of MNPs and PLL-MNPs

**Fig. 1:**
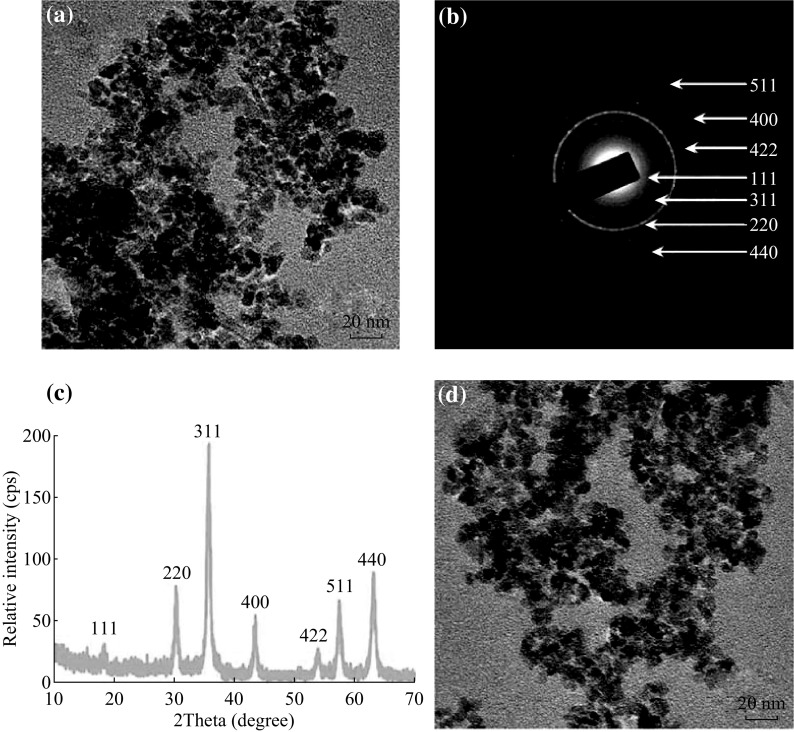
**a** TEM image, **b** EDX results, and **c** XRD pattern of MNPs. **d** TEM image of PLL-MNPs

Figure 1a shows a TEM image of the MNPs indicating the synthesis of nanosized MNPs. The diameter of a single MNP was approximated to be in the range of 10–15 nm. The electron diffraction pattern (Fig. 1b), corresponding to bright-field images, showed that these particles consisted of MNPs. This was also confirmed by their XRD patterns (Fig. 1c), which proved that their crystalline nature and the peaks matched standard γ-Fe_2_O_3_ reflections; no α-Fe_2_O_3_ phase was observed, although the product was brown. Figure 1d shows the TEM image of the prepared PLL-MNPs, indicating that the PLL modification did not significantly affect the nanosized structure or morphology, consistent with observations in previous studies [41, 42].

**Fig. 2:**
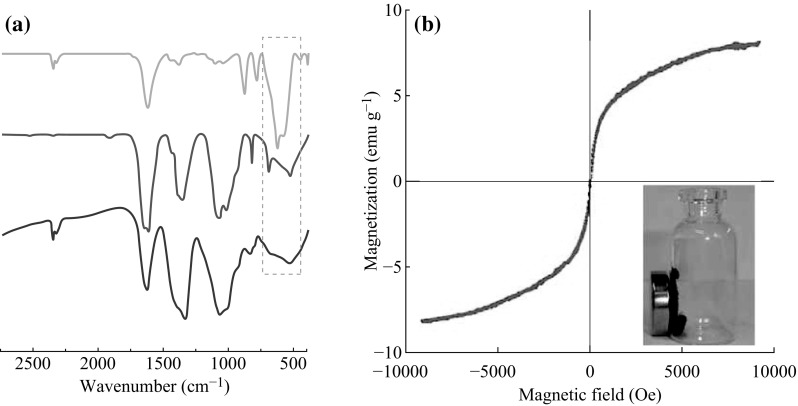
**a** FT-IR spectra of MNPs (*top*), PLL-MNPs (*middle*), and pure PLL (*bottom*). **b** The magnetic loop of PLL-MNPs at 300 K

FT-IR spectra of the MNPs and PLL-MNPs confirmed that MNPs were successfully modified by PLL, because the characteristic adsorption band of Fe–O was observed at 554 cm^−1^ (Fig. 2a). The magnetization curve of the prepared PLL-MNPs demonstrated a symmetrical hysteresis loop (Fig. 2b), which is characteristic of superparamagnetism. In other words, the PLL-MNPs become magnetized in the presence of a magnetic field and, once the field is removed, only a minimal residual magnetization remains within the particles [43]. This characteristic makes the prepared PLL-MNPs ideal as magnetic target carriers. Moreover, the saturation magnetization and coercivity of the PLL-MNPs were also estimated to be approximately 8.1 emu g^−1^ and 12.5 Oe, respectively.

### Labeling of A549 Lung Cancer Cells with the PLL-MNPs

**Fig. 3:**
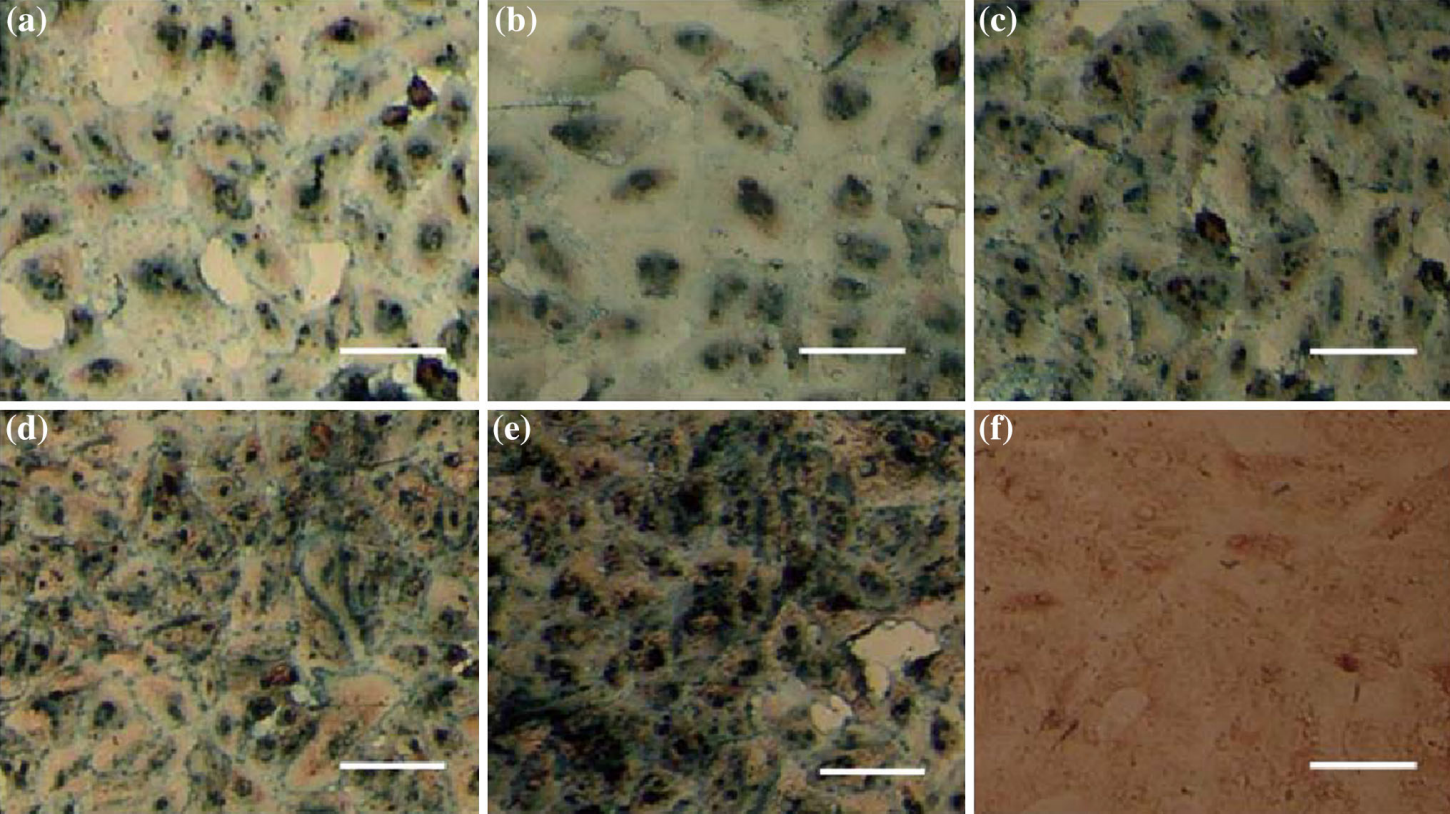
Prussian blue staining of A549 lung cancer cells labeled with PLL-MNPs at different concentrations (25, 50, 100, 200, and 400 µg mL^−1^ for **a**, **b**, **c**, **d**, and **e**, respectively) and **f** unlabeled A549 cells used as controls. *Scale bar* 100 µm

A549 cells were treated with the prepared magnetic PLL-MNPs for cell labeling. The Prussian blue method was employed to detect iron within the treated cells through the reduction of ferric iron into the ferrous state as indicated by a blue precipitate [44]. The results showed that the blue precipitate formed, indicating the presence of the PLL-MNPs within A549 cells (Fig. 3).

### Proliferation Capacity of PLL-MNP-Labeled A549 Lung Cancer Cells

**Fig. 4:**
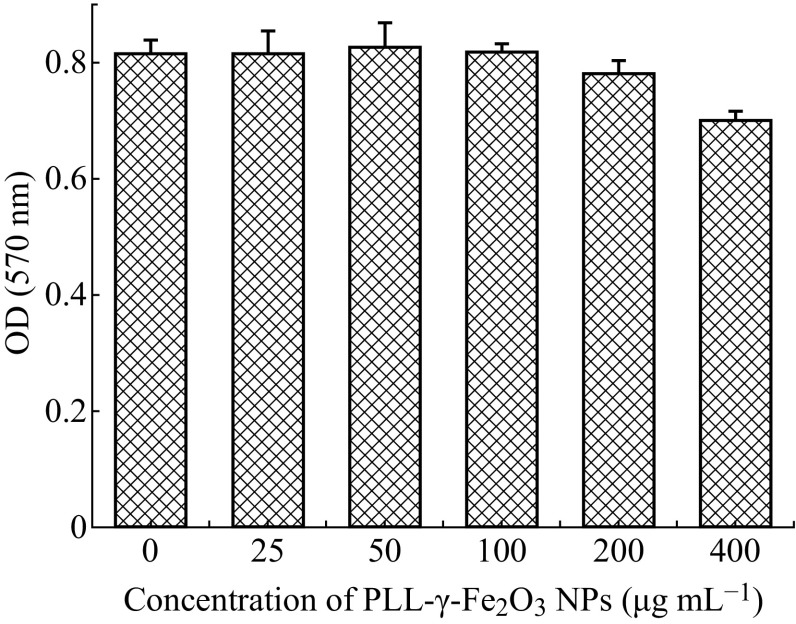
The temporal growth state of the PLL-MNP-labeled A549 lung cancer cells incubated in RPMI-1640 medium, and the untreated counterparts were used as controls

The MTT assay is a simple colorimetric method used to measure cell proliferation [35, 45]. A549 cells were incubated with the PLL-MNPs at different concentrations varying from 25 to 400 μg mL^−1^; unlabeled A549 cells were used as the control. The results indicated that the A549 cells labeled with PLL-MNPs at low concentrations, ranging from 25 to 200 μg mL^−1^, generally exhibited a comparable proliferation ability in contrast with the unlabeled cells. However, A549 cells labeled with the PLL-MNPs at a concentration of 400 μg mL^−1^ showed a reduced proliferation potential (Fig. 4). Therefore, the results indicate that the proliferation capacity of the A549 cells was not obviously affected by magnetic labeling of the PLL-MNPs at low concentrations.

### Cell Viability of PLL-MNP-Labeled A549 Lung Cancer Cells

**Fig. 5:**
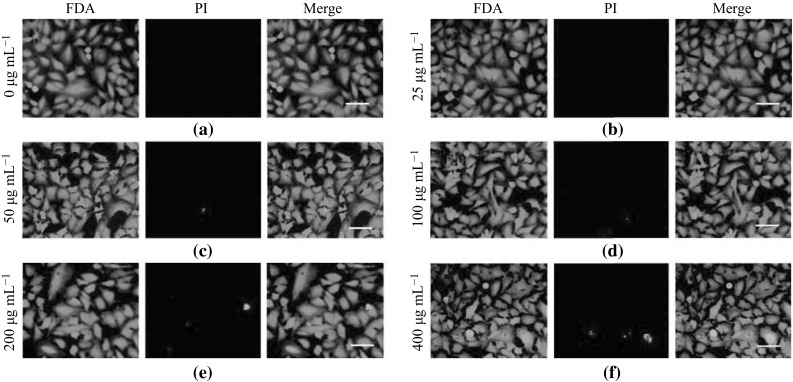
Cellular viability detection of the PLL-MNP-labeled A549 lung cancer cells using PI and FDA double staining. **a** Untreated A549 cells were used as controls. **b–f** Fluorescence images illustrated the A549 cells treated with various concentrations of PLL-MNPs (25, 50, 100, 200, and 400 µg mL^−1^) for 48 h. *Scale bar* 100 µm

The PI and FDA double-staining protocol was used to detect the cell viability of PLL-MNP-labeled A549 cells. PI is a DNA-binding fluorescent dye that only enters dead or dying cells with damaged or leaky membranes; therefore, it is used as a marker for apoptotic and necrotic cells [39, 40]. On the other hand, FDA, which stains cells with intact membranes, produces a bright green fluorescence [39, 40]. The results showed that the PLL-MNP-labeled A549 cells exhibited intact viability compared with the unlabeled A549 cells (Fig. 5).

### Actin Cytoskeleton of PLL-MNP-Labeled A549 Lung Cancer Cells

**Fig. 6:**
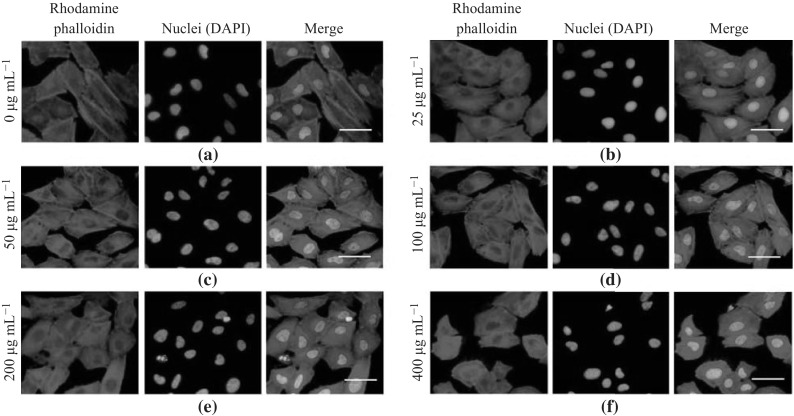
Immunofluorescent staining of the actin cytoskeleton of the PLL-MNP-labeled A549 lung cancer cells. **a** Untreated A549 cells were used as controls. **b–f** The A549 cells treated with different concentrations of PLL-MNPs (25, 50, 100, 200, and 400 µg mL^−1^) were cultured for 48 h in an RPMI-1640 medium. Cell nucleus was located by counterstaining with DAPI (*blue*). *Scale bar* 100 µm. (Color figure online)

Cytoskeleton microfilaments are commonly involved in normal cell attachment and morphology [46, 47]; thus, the altered appearance of actin filaments in the PLL-MNP-labeled A549 cells could indicate that the PLL-MNPs are highly cytotoxic. Rhodamine phalloidin specifically binds to the polymerized F-actin with a high affinity. The immunocytochemical staining of the actin cytoskeleton in the PLL-MNP-labeled A549 cells is shown in Fig. 6. The results show that the A549 cells labeled with the PLL-MNPs at concentrations ranging from 25 to 200 μg mL^−1^ exhibited normal cytoskeleton organization compared with their untreated counterparts. However, when the concentration of the PLL-MNPs increased to 400 μg mL^−1^ in the labeled cells, the cytoskeleton was slightly affected as observed by a relatively small and round morphology and a slight disorganization of F-actin. Therefore, these data indicate that the PLL-MNPs, at appropriate concentrations, could be used for the cell labeling of human A549 lung cancer cells.

### Effects of PLL-MNP Labeling on the Cell Cycle of A549 Cells

**Fig. 7:**
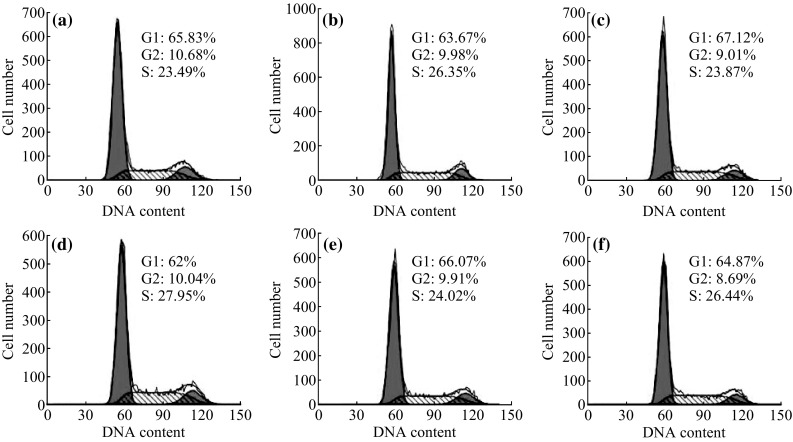
**a** Cell cycle distribution of the examined cells. Unlabeled A549 cells were used as controls. **b–f** The A549 cells labeled with different concentrations of PLL-MNPs (25, 50, 100, 200, and 400 µg mL^−1^) were cultured for 48 h

The effects of PLL-MNP treatment on the cell cycle distribution of A549 cells were analyzed using PI staining and a flow cytometer. The results showed that the DNA composition in S-phase is 23.49 % for the unlabeled A549 cells (Fig. 7a) and that the DNA composition in S-phase of the PLL-MNP-labeled A549 cells with different concentrations of PLL-MNPs is 26.35, 23.87,27.95,24.02, and 26.44 % (Fig. 7b–f). Also, there are no obvious differences in other phases. The results indicated that PLL-MNP treatment did not affect distribution of cell cycle for A549 cells.

### Apoptosis Assay

Cell apoptosis is a distinct process of programed cell death and plays an important role in carcinogenesis and cancer treatment. Apoptosis is generally characterized by distinct morphological characteristics of related cells, which usually include blebbing, cell shrinkage, nuclear fragmentation, chromatin condensation, and chromosomal DNA fragmentation [48, 49]. In contrast, tumor cells are characterized by their ability to bypass apoptosis. Thus, apoptosis is commonly used as a vehicle for targeted cell treatment in cancer therapy [50, 51]; medications used to induce apoptosis of tumor cells are a major focus of cancer treatment.

**Fig. 8:**
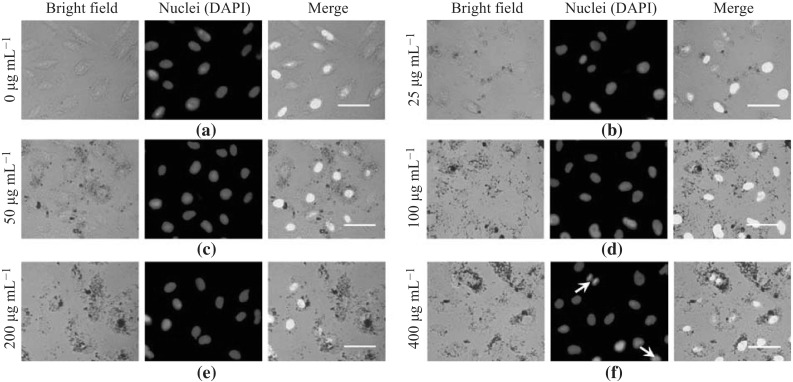
Fluorescent staining of PLL-MNP-treated A549 lung cancer cells. **a** Untreated A549 cells were used as controls. **b–f** Fluorescence images illustrated the A549 cells treated with different concentrations of PLL-MNPs (25, 50, 100, 200, and 400 µg mL^−1^). *Scale bar* 100 µm

In the present study, A549 cells were treated with different concentrations of the PLL-MNPs and then stained with fluorescent dye Hoechst H33258, a bisbenzimide dye that binds to AT-rich regions of DNA allowing the detection and relative quantitation of apoptotic cell DNA [52, 53]. To visualize the nuclear fragmentation of apoptotic cells, the typical changes of treated A549 cells were related with chromatin condensation, nuclear peripheral aggregation, and nuclear fragmentation using an inverted fluorescence microscope (Fig. 8). The results show that nuclear fragmentation and chromatin condensation were detected in the treated cells at high PLL-MNP concentrations.

## Conclusions

In the present study, the PLL-MNPs were fabricated with excellent superparamagnetic properties and were used to label human A549 lung cancer cells. The effects of magnetic labeling on the biological behavior of A549 lung cancer cells were also analyzed. The results showed that, in comparison with previous studies, the fabricated PLL-MNPs could enhance the cellular uptake in A549 lung cancer cells, the low concentrations of the PLL-MNPs used to label cells did not affect the cell proliferation, viability, and cytoskeleton (F-actin) organization in comparison with unlabeled counterparts, and that cell apoptosis was slightly affected by high concentrations of the PLL-MNPs. In general, the present study provides the detailed insights into the efficient magnetic IONP labeling of A549 lung cancer cells and sheds light on the effects of magnetic labeling on the biological behavior of these cells. The results presented herein provide a new perspective for the application of magnetic IONPs in the cell-targeted labeling and noninvasive tracking behavior of A549 lung cancer cells, as well as offering a promising approach for cell-based lung cancer treatment.
